# Nitric oxide agents impair insulin-mediated signal transduction in rat skeletal muscle

**DOI:** 10.1186/1471-2091-7-17

**Published:** 2006-05-27

**Authors:** Simone Badal, Paul D Brown, Dalip Ragoobirsingh

**Affiliations:** 1 Department of Basic Medical Sciences, Section of Biochemistry, The University of the West Indies, Kingston, Jamaica

## Abstract

**Background:**

Evidence demonstrates that exogenously administered nitric oxide (NO) can induce insulin resistance in skeletal muscle. We have investigated the modulatory effects of two NO donors, S-nitroso-N-acetyl-D, L-penicillamine (SNAP) and S-nitrosoglutathione (GSNO) on the early events in insulin signaling in rat skeletal myocytes.

**Results:**

Skeletal muscle cells from 6–8 week old Sprague-Dawley rats were treated with SNAP or GSNO (25 ng/ml) in the presence or absence of glucose (25 mM) and insulin (100 nM). Cellular insulin receptor-β levels and tyrosine phosphorylation in IRS-1 were significantly reduced, while serine phosphorylation in IRS-1 was significantly increased in these cells, when compared to the insulin-stimulated control. Reversal to near normal levels was achieved using the NO scavenger, 2-(4-carboxyphenyl)-4, 4, 5, 5-tetramethylimidazoline-1-oxyl 3-oxide (carboxy-PTIO).

**Conclusion:**

These data suggest that NO is a potent modulator of insulin-mediated signal transduction and may play a significant role in the pathogenesis of type 2 diabetes mellitus.

## Background

Nitric oxide (NO) is an important bioactive molecule that regulates a variety of normal physiological functions and is involved in the mediation of several pathologic processes [1,2]. It is a short-lived free radical gas and endogenous signalling molecule produced by the intracellular enzyme NO synthase [2]. NO drugs are useful in the treatment of several disorders, and are generally indicated in cases of NO insufficiency. Previously, we have established that exogenous NO (from NO-releasing drugs) inhibited in vivo insulin binding to its receptor on erythrocytes and mononuclear leukocytes [3], and in vitro glucose uptake in skeletal muscle cells [4] and adipocytes (unpublished results). Skeletal muscle is an important target for insulin action and insulin resistance here is a characteristic feature of type 2 diabetes [5].

Insulin is the principal hormone controlling blood glucose and acts by stimulating glucose influx and metabolism in muscle and adipocytes and inhibiting gluconeogenesis by the liver [6]. Insulin action is mediated through the insulin receptor, a transmembrane glycoprotein with intrinsic protein tyrosine kinase activity [6]. The level of tyrosine kinase reflects the serum concentration of insulin and appears to mediate the insulin response through tyrosine phosphorylation of the receptor itself and substrates like insulin receptor substrate (IRS)-1 [7-9]. Phosphorylation of IRS-1 on multiple tyrosine residues creates an active signalling complex by recruiting various proteins, including phosphatidyl 3 kinase (PI3K), Grb2, SHP2, among others [10,11]. Dys-regulation of the insulin receptor and IRS-1 proteins are usually associated with type 2 diabetes [12,13], occasioned by proteasome-mediated degradation [14,15], phosphatase-mediated dephosphorylation [16,17] or kinase mediated serine/threonine phosphorylation [18]. Our current objective was to characterize the *in vitro *effects of exogenous NO generated by S-nitroso-N-acetylpenicillamine (SNAP) and S-nitrosoglutathione (GSNO) on the cellular levels of insulin receptor-β (IR-β), and phosphorylated tyrosine and serine residues in isolated rat skeletal myocytes.

## Results

### Nitric oxide released from drugs

**Figure 1:**
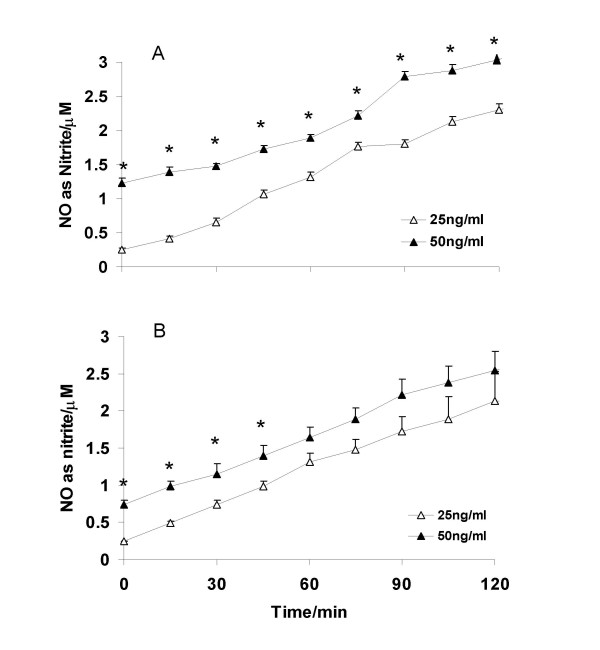
Nitric oxide (NO) released from (A) SNAP and (B) GSNO at 25 and 50 ng/ml. Values are expressed as means ± SEM; * p < 0.05 vs the drug at 25 ng/ml.

**Figure 2:**
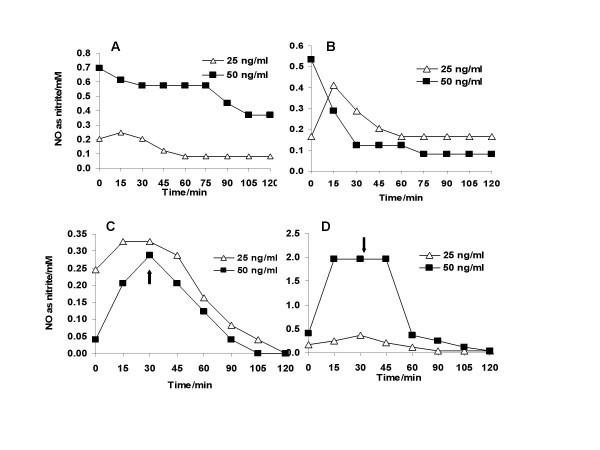
Nitric oxide (NO) released from (A and C) SNAP and (B and D) GSNO at 25 and 50 ng/ml in the presence of the NO-scavenger, carboxy-PTIO, added at time 0 min (A and B) and after 30 min (C and D).

Figure 1 shows the concentration-dependent increase in nitric oxide (measured as nitrite) released from SNAP and GSNO in aqueous solution. In all cases there was a gradual increase in NO released, with a greater amount of NO being released from drugs at the higher concentration. Carboxy-PTIO, when added either at the start of the experiment or after 30 min resulted in a sharp decline in the amount of NO released from either drug (fig. 2).

### Effect of NO released from SNAP and GSNO on IR-β expression

**Figure 3:**
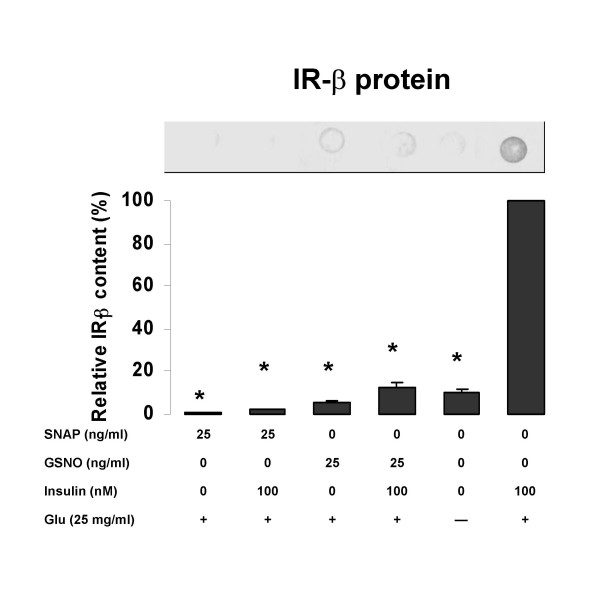
The effects of NO donors on content of IR-β in rat skeletal myocytes. Rat myocytes were treated with 25 ng/ml of SNAP or GSNO, in the presence the indicated concentrations of insulin, with or without 25 mg/ml glucose (Glu). Values are expressed as means ± SEM; * p < 0.05 vs the insulin-stimulated control (100 mM insulin). One representative blot is shown.

Figure 3 illustrates the inhibitory effects of NO released from SNAP and GSNO on IR-β expression in isolated rat skeletal myocytes. Incubation with SNAP significantly decreased expression of IR-β compared to the insulin-stimulated control by 74 – 99 % (p < 0.01). Similar results were obtained for GSNO; however, these reductions were not as dramatic, but were of the order of the unstimulated negative control. For both drugs, there was a slight increase in the expression of IR-β in cells treated with the NO donor and insulin when compared to those treated with the NO donor alone (p > 0.05); in the case of GSNO, the increase approached significance.

### Effect of NO released from SNAP and GSNO on tyrosine phosphorylation of IRS-1

**Figure 4:**
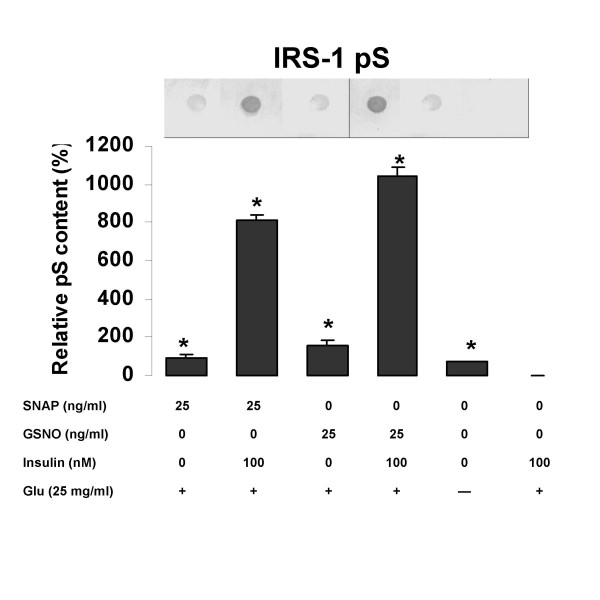
The effects of NO donors on tyrosine phosphorylation (pY) in rat skeletal myocytes. Rat skeletal myocytes were treated with 25 ng/ml of SNAP or GSNO, in the presence the indicated concentrations of insulin, with or without 25 mg/ml glucose (Glu). Values are expressed as means ± SEM; * p < 0.05 vs the insulin-stimulated control (100 mM insulin). One representative blot is shown.

Tyrosine phosphorylation of IRS-1 was significantly reduced in the presence of SNAP and GSNO (fig. 4). Incubation with SNAP or GSNO significantly reduced the levels of IRS-1 pY in these cells compared to the insulin-stimulated control (p < 0.01). There was a 20% increase in the level of tyrosine phosphorylation in the presence of insulin in cells treated with either drug (p < 0.05). However, there was no difference between the drugs whether insulin was present or absent.

### Effect of NO released from SNAP and GSNO on serine phosphorylation in IRS-1

**Figure 5:**
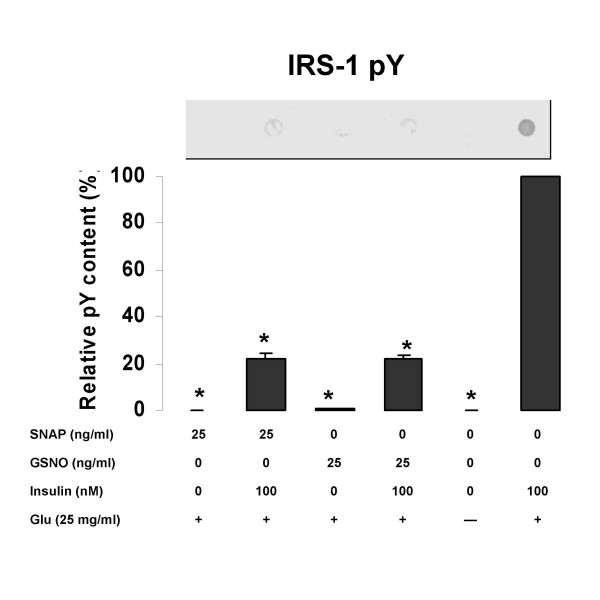
The effects of NO donors on serine phosphorylation (pS) in rat skeletal myocytes. Rat skeletal myocytes were treated with 25 ng/ml SNAP or GSNO, in the presence the indicated concentrations of insulin, with or without 25 mg/ml glucose (Glu). Values are expressed as means ± SEM; * p < 0.05 vs the insulin-stimulated control (100 mM insulin). One representative blot is shown.

Figure 5 shows the effect of SNAP and GSNO on serine phosphorylation in IRS-1. Unlike the trends observed for tyrosine phosphorylation, serine phosphorylation was significantly increased in the presence of both drugs, whether insulin was present or not (p < 0.05). GSNO was significantly more effective than SNAP in increasing serine phosphorylation in the absence or presence of insulin.

**Figure 6:**
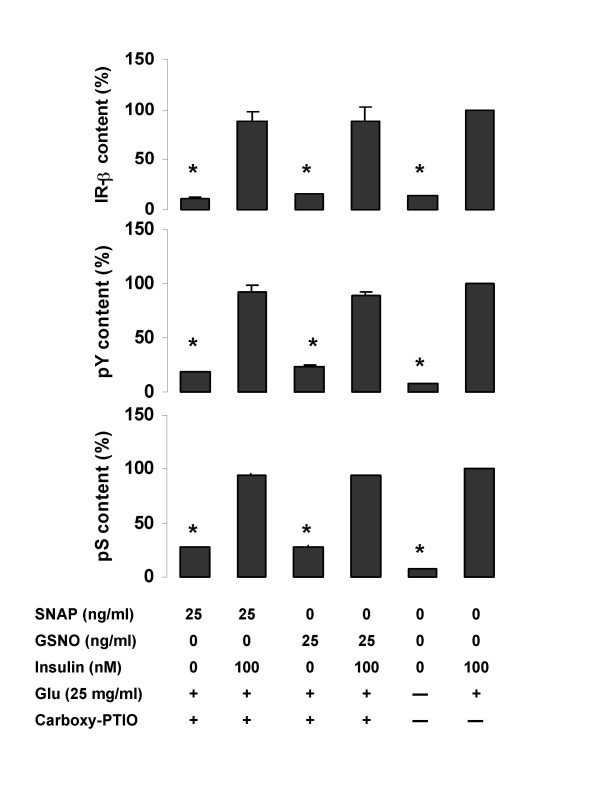
The effects of a NO scavenger, carboxy-PTIO and NO donors on content of IR-β (A), tyrosine phosphorylation (B) and serine phosphorylation (C) in rat skeletal myocytes. Rat skeletal myocytes were treated with the NO-scavenger, carboxy-PTIO (0.1 μM) with 25 mg/ml of GSNO or SNAP in the presence the indicated concentrations of insulin, with or without 25 mg/ml glucose (Glu). Values are expressed as means ± SEM; * p < 0.05 vs the insulin-stimulated control (100 mM insulin).

We tested whether the NO scavenger, carboxy-PTIO was able to reverse the effect of NO-mediated reduction in expression of IR-β, and extent of tyrosine and serine phosphorylation in the skeletal myocytes. We found a near normal expression of IR-β in myocytes co-treated with carboxy-PTIO and SNAP or GSNO in the presence of insulin (fig. 6A). These were not significantly different from the respective controls with insulin (p > 0.3). Similar trends were observed for tyrosine and serine phosphorylation when the cells were exposed to the drugs in the presence of the NO scavenger (fig. 6B and 6C).

## Discussion

The present study clearly demonstrates that exogenously administered nitric oxide reduced the expression of the insulin receptor β subunit and the levels of tyrosine phosphorylation while increasing serine phosphorylation in rat skeletal myocytes. These effects are expected to significantly impair the insulin-mediated signal transduction pathway leading to glucose uptake and metabolism, and they confirm that pathophysiologically relevant concentrations of NO are able to affect several points in the metabolic pathway mediated by insulin. We found that SNAP and GSNO release NO in a quantitative manner, and in the absence of a quenching agent, produce quantities of NO which can diffuse across the cell membranes and disrupt normal cellular processes. There are two possible modes for the release of NO from SNAP and GSNO in *in vitro *systems. First, decomposition of nitrosothiols (RS-NOs) can be catalyzed by intrinsic copper or iron ions forming the respective thiol and NO [19,20]. Second, enzymatic NO release from RS-NOs can occur at the cell surface [21,22], forming one electron reduction of RS-NOs and resulting in the release of a neutral NO molecule [23]. The latter appears to be the more efficient process of RS-NO decomposition, and because SNAP generates more NO than GSNO [24,25], it is expected to have a greater overall effect than GSNO.

In this study, we were able to completely abrogate the deleterious effects of both drugs with the NO scavenger, which suggests that the effects of SNAP and GSNO were related to the generation of NO and not to a non-specific effect of the donors. Further, these results suggest that the effects of NO impairment might be reversible if treated early, and before the pathologic sequelae associated with diabetes is evidenced.

Insulin action is initiated through its binding to the cell-surface receptor, initiating a series of signal transduction reactions, which stimulate various effectors to produce its physiological effects. Therefore, impairment of insulin signal transduction results in attenuation of insulin action and leads to insulin resistance resulting in type 2 diabetes mellitus. Because the molecular mechanisms of insulin resistance are still being elucidated, it is indispensable to establish in vitro models of basal and insulin-mediated signal transduction to clarify these mechanisms and suggest treatments where appropriate.

Skeletal muscle is responsible for about 75% of whole body glucose metabolism, and insulin resistance is a characteristic feature of individuals with type 2 diabetes mellitus [26]. A number of intracellular defects in insulin action in muscle have been described, including decreased glucose transport [27,28] and glucose phosphorylation [29] and diminished glycogen synthase activity [30]. A similar effect is observed in rodent model systems [31,32]. In this study we noted that acute treatment of skeletal myocytes by either GSNO or SNAP resulted in significantly reduced content of available IR-β for participating in insulin-mediate signal transduction. This could be a possible explanation for the decrease in insulin binding and insulin receptor sites observed in mononuclear leukocytes and erythocytes treated with these NO donors [3]. Further, recent findings highlight the involvement of exogenous NO in S-nitrosation of IR-β in isolated rat muscle, with the associated reduction in insulin-induced insulin receptor autophosphorylation and tyrosine kinase activity [33]. While these authors found the reduction after chronic exposure to GSNO, we found similar reductions in IR-β expression after acute exposure, which strongly suggests that the reduction observed, might not be due to S-nitrosation. This acute reduction in expression is expected to be associated with a marked reduction in insulin binding and signalling, which would translate into reduced glucose transport and glycogen storage in isolated muscle treated with the NO donors [4]. We noted an additive effect of the drugs on IR-β expression in the presence of insulin, although the levels of expression were not significantly different from the untreated controls. While this increase might have not been expected, it has been previously reported in relation to glucose uptake in the presence of NO donors and insulin [34].

Prior to the publication by Carvalho-Filho and co-workers [33], the postulated mechanisms for insulin resistance involved either increased phosphotyrosine phosphatase activity (effectively reduced tyrosine phosphorylation) or increased serine phosphorylation of IRS proteins [16,18], and only increased or preferential serine phosphorylation of IRS proteins had been linked to insulin resistance mediated by their degradation [35,36]. It is well established that changes in the level of phosphorylation at any of the possible sites on these proteins could potentially alter their ability to bind and activate the various downstream effectors in the insulin-mediated signal transduction pathway [6]. It is our view that S-nitrosation is secondary to serine phosphorylation as the means whereby NO mediates insulin resistance in skeletal muscle. This is based on the fact that IRS degradation effected by S-nitrosation occurs only after chronic exposure to the NO donor [33], unlike proteasome-mediated degradation subsequent to serine phosphorylation [37]. In this study we found that the NO donors caused decreased tyrosine and increased serine phosphorylation in IRS-1 in skeletal myocytes. Although the reduction in tyrosine phosphorylation may be due to preferentially serine phosphorylation in these molecules, we cannot rule out the possibility that tyrosine nitration may also be occurring and be contributing to the NO-mediated insulin resistance in these cells. Further, while a reduction in tyrosine phosphorylation in IRS-1 per se does not reduce IRS-1 content [38], it will result in insulin resistance in skeletal muscle. Because skeletal muscle is the largest insulin-sensitive organ in humans, NO-induced insulin resistance in this tissue will have a major impact on whole body glucose homeostasis, especially in patients who are obese or need to take NO drugs for prolonged periods. An equally plausible explanation for the reduced tyrosine phosphorylation in IRS-1 could be due to the lower amount of insulin receptor that is being expressed, due to the action of NO.

Serine phosphorylation of IRS proteins has been established a means to terminate insulin action. However, this has been found to commence after tyrosine phosphorylation of IRS proteins which trigger insulin signalling [36], based on their finding that phosphorylation of serine 408 was increased after insulin treatment, and was sensitive to wortmanin (suggesting that the kinase is a downstream effector of PI3-K). In addition to the fact that the phosphorylation of serine residues within IRS proteins marks them for degradation, there is further evidence that other processes may be involved. For example, serine phosphorylation can induce the dissociation of IRS proteins from the insulin receptor [39], or hinder tyrosine phosphorylation sites [40], or release the IRS proteins from intracellular complexes that maintain them in close proximity to the receptor [41], or turn IRS proteins into inhibitors of the IR kinase [18]. Therefore, it is possible that multiple mechanisms can contribute to insulin resistance and thus impair insulin-mediated signal transduction, and reversal of one of them can improve insulin action, as have been previously reported [16,18].

It is widely believed that phosphorylation of a single serine residue in IRS-1 might not be sufficient to inhibit tyrosine phosphorylation of IRS-1 and uncouple IR-IRS complexes, although it could be a target fro phosphorylation by IRS kinases activated only by selective inducers of insulin resistance. Some of these serine residues phosphorylated are catalyzed by a number of kinases, which may in fact be activated by insulin [42-48], which might explain our observations that there was an additive effect of the drugs on serine phosphorylation in the presence of insulin.

## Conclusion

From the results presented herein, it is clear that NO released from the NO donors has a negative effect on IR-β expression and tyrosine phosphorylation of IRS-1 and a positive effect on serine phosphorylation of IRS-1 in rat skeletal myocytes. These effects would have significant bearing on initial transduction and the availability of downstream effectors of the insulin signal. Taken together, these results indicate a direct role of NO in the impairment of insulin-mediated signal transduction in skeletal muscle, and possibly in the pathogenesis of type 2 diabetes mellitus.

## Methods

### Chemicals and reagents

Anti-insulin receptor-β (anti-IR-β), anti-IRS-1, anti-phosphotyrosine (anti-pY), anti-phosphoserine (anti-pS) and associated alkaline phosphatase conjugates were obtained from Chemicon International Inc. (Temecula, CA, USA). All biochemicals and enzymes were of molecular biology grade and were purchased from commercial suppliers.

### Preparation of dissociated cells

Female and male Sprague-Dawley rats (6 – 8 weeks old weighing 200 – 300 g) were housed at the University of the West Indies Preclinical Animal House, Mona, Jamaica, with free access to water and food. Food was withdrawn on the evening prior to experimentation and rats were euthanatized using diethyl ether. The Ethics Committee of the University of the West Indies/University Hospital of the West Indies approved the experiments involving animals and method of euthanasia.

Approximately 5–10 g of skeletal muscle tissue were aseptically removed, washed 2–3 times with sterile phosphate buffered saline (PBS, pH 7.2) and minced using a sterile scalpel. Skeletal myocytes were isolated using a modification of the method by Freshney [49]. Briefly, minced tissue was incubated in Krebs' Ringer Bicarbonate (KRB) containing 200 U/ml collagenase, 10% bovine serum albumin (BSA) and 1x penicillin/streptomycin/neomycin (PSN) (Sigma, St. Louis, MO, USA) at 37°C in a humidified incubator containing 95% air and 5% CO_2_. Dissociated cells were collected by centrifugation at 100 rpm for 5 min, washed twice in 5 ml KRB buffer containing 2.5 mM glucose and 2% BSA and resuspended in 20–30 ml KRB without any additives. Viability of dissociated cells was verified using Trypan blue.

### Treatments of dissociated cells

After an incubation period of 30 min, cells (1 ml packed cells/1.5 ml Eppendorf tube) were treated with 25 ng/ml SNAP or GSNO (Sigma), in the absence or presence of 100 nM insulin at 37°C for 1 hr. Nitric oxide released from the drugs was determined as nitrite using the Greiss reagent (BDH, Poole, UK). Experiments with 25 ng/ml GSNO and SNAP were repeated in the presence of 0.1 μM of the NO scavenger, 2-(4-carboxyphenyl)-4, 4, 5, 5-tetramethylimidazoline-1-oxyl 3-oxide (carboxy-PTIO; Sigma).

### Immunoblot analysis

Cells were washed and solubilized in 750 μl of lysis buffer (20 mM HEPES, 150 mM NaCl, 1 % Triton X-100, 1 mM aprotinin, 0.2 mM leupeptin, 1 mM phenymethylsulfonyl fluoride [PMSF], 1 mM sodium orthovanadate) for 30 min at 4°C. Detergent-insoluble material was sedimented by centrifugation at 12,000 g for 10 min at 4°C. The protein content of cell lysates was determined using the Bradford method [50]. Cell lysate proteins (30–50 μg) or 50 μg of immunoprecipitated IRS-1 were vacuum blotted onto poly(vinylidene difluoride) (PVDF) membrane using a manifold apparatus (Scie-Plas, Warwickshire, UK). Immunoprecipitation was performed by incubating the lysates with anti-IRS-1 at 4°C for 3 h. Immune complexes were collected with protein G agarose (Sigma) for 1.5 h at 4°C, washed, and solubilized in lysis buffer.

Membranes were analyzed using the Protein detector Western Blot Kit BCIP/NBT System (Kirkegaard and Perry Laboratories [KPL], Gaithersburg, MD, USA). After blocking, membranes were probed with anti-IR-β (1:500) for lysates, or anti-pY (1:1000), or anti-pS (1:1000) antibodies for solubilized immunoprecipitates, according to the manufacturer's recommendations. Chromogenic detection of the bound antibodies was done using secondary antibodies conjugated to alkaline phosphatase, as described by the manufacturer (KPL, Gaithersburg, MD, USA). Densitometric analysis of the blots was carried out using NIH Image J programme for PC [51].

### Statistical analysis

Statistical analysis was performed using either unpaired Student's t test or ANOVA (Fisher, multiple comparisons), as applicable. Values are presented as means ± S.E.M. of 3–6 observations, and differences among means were considered significant at p < 0.05.

## Abbreviations

Carboxy-PTIO, 2-(4-carboxyphenyl)-4, 4, 5, 5-tetramethylimidazoline-1-oxyl 3-oxide; GSNO, S-nitrosoglutathione; IR-β, insulin receptor β; NO, nitric oxide; pS, phosphorylated serine; pY, phosphorylated tyrosine; PSN, 1x penicillin/streptomycin/neomycin; PVDF, poly(vinylidene difluoride); SNAP, S-nitroso-N-acetylpenicillamine;

## Competing interests

The author(s) declare that they have no competing interests.

## Authors' contributions

SB was involved in the literature search, study design, experimentation, data collection and interpretation; PDB conceived of the study, and was involved in literature search, statistical analysis, data interpretation and manuscript preparation; DR was involved in study design and data interpretation. All authors read and approved the final manuscript.
